# Oral Prevalence of *Selenomonas noxia* Differs among Orthodontic Patients Compared to Non-Orthodontic Controls: A Retrospective Biorepository Analysis

**DOI:** 10.3390/pathogens13080670

**Published:** 2024-08-08

**Authors:** Kyle Hodges, Payton Famuliner, Karl Kingsley, Katherine M. Howard

**Affiliations:** 1 Department of Advanced Education in Pediatric Dentistry, School of Dental Medicine, University of Nevada-Las Vegas, 1700 West Charleston Blvd, Las Vegas, NV 89106, USA; 2 Department of Clinical Sciences, School of Dental Medicine, University of Nevada-Las Vegas, 1700 West Charleston Blvd, Las Vegas, NV 89106, USA; 3 Department of Biomedical Sciences, School of Dental Medicine, University of Nevada-Las Vegas, 1001 Shadow Lane, Las Vegas, NV 89106, USA; katherine.howard@unlv.edu

**Keywords:** *Selenomonas noxia*, oral prevalence, orthodontic appliance, saliva screening, qPCR

## Abstract

The oral microbial flora may be significantly altered by orthodontic therapy and the use of fixed orthodontic brackets. Most orthodontic research has focused on cariogenic pathogens, while some evidence has demonstrated an increase in many known periodontal pathogens. However, little is known about the prevalence of the Gram-negative periodontal pathogen *Selenomonas noxia* (SN) among these patients. Using an existing saliva biorepository, *n* = 208 samples from adult and pediatric orthodontic and non-orthodontic patients were identified and screened for the presence of SN using qPCR and validated primers. In the pediatric study sample (*n* = 89), 36% tested positive for the presence of SN, with orthodontic patients comprising more SN-positive samples (87.5%) than SN-negative samples (78.9%), *p* = 0.0271. In the adult study sample (*n* = 119), SN was found in 28.6%, with orthodontic patients comprising 58.8% of positive samples and only 28.2% of negative samples (*p* < 0.0001). These data demonstrated that both pediatric and adult orthodontic patients exhibited higher prevalence of SN compared with age-matched non-orthodontic controls. As this microorganism is associated not only with periodontal disease but also long-term health issues such as obesity, more research is needed regarding the factors that increase the prevalence of this microbe.

## 1. Introduction

Many factors have the potential to alter the oral microbiome, including the use of fixed orthodontic brackets as part of orthodontic therapy [1,2]. Although many studies have demonstrated significant changes in the oral microbiome during orthodontic therapy, systematic reviews of fixed appliances, such as conventional (CBs), passive self-ligating (PSLBs), or active self-ligating (ASLBs) brackets, have demonstrated that most clinical and patient-specific outcomes (including changes in microbial growth and biofilm formation) did not differ significantly between these types [3,4]. Due to the development of white spot lesions (WSLs) caused by microbial growth in and around the tooth bonding site, many studies have focused on methods of detecting and preventing the development of WSL through increased oral hygiene and other prevention methods [5,6].

Most of the research in this area has focused on the increase in prevalence of cariogenic pathogens, which markedly increase in many cases of orthodontic treatment [7,8,9]. The Gram-positive microorganisms *Streptococcus mutans* (SM) and *Lactobacillus acidophilus* (LA) have been the subject of many studies, as these cariogenic microorganisms are highly associated with the development of WSL and adverse oral health outcomes among orthodontic patients [10,11,12]. Additional Gram-positive cariogenic bacteria have been identified, including *Streptococcus sobrinus* and *Scardovia wiggsiae*, which may also be affected by orthodontic treatment and the increased incidence of caries and WSL [13,14,15].

However, some evidence has suggested that other alterations during orthodontic therapy may include gingivitis, juvenile periodontitis, and an increase in many known periodontal pathogens [16,17]. These include the most widely studied periodontal disease-associated microorganisms, such as *Porphyromonas gingivalis* (PG), *Fusobacterium nucleatum* (FN), *Aggregatibacter actinomycetemcomitans* (AA), *Prevotella intermedia* (PI), and *Tannerella forsythia* (TF) [18,19,20]. However, some studies have revealed that another important Gram-negative periodontal pathogen, *Selenomonas noxia* (SN), may also be influenced by orthodontic treatment and therapy [21].

Some research has identified SN as an early transitional microbe that increases significantly among patients with gingivitis who subsequently develop periodontal disease [22,23]. In fact, SN has also been identified in the subgingival microbiota of pregnant and postpartum patients that developed pregnancy-induced gingivitis and periodontal disease, as well as other periodontal disease-associated conditions, such as Down’s and Papillon–Lefevre syndrome [24,25,26]. Importantly, SN has also been identified among adolescents with gingivitis and periodontitis—an age group that comprises a large percentage of those undergoing orthodontic treatment and therapy [27,28]. These studies build on early studies that identified SN as part of an important panel of microbial species that could indicate the onset of periodontal disease [29,30,31].

The interest in SN has grown recently, as this microorganism has been not only associated with periodontal disease but has also been linked with other negative long-term health outcomes, including obesity [32,33]. This may be due to the ability of SN to metabolize indigestible fibers and cellulose, thereby releasing dietary calories that increase the potential for host patients to become overweight or obese [34,35]. In fact, new data suggest that interventions which modulate the levels and prevalence of this microorganism may be strongly associated with significant losses in weight and reductions in body mass index [36,37,38].

Due to the recent discovery of this microorganism and these associated health problems, including periodontal disease and obesity, few studies have evaluated the effect of orthodontic treatment with the prevalence of this microbe [21,39]. Based upon the relatively limited amount of evidence regarding the prevalence of this microorganism and the potential association with orthodontic treatment, the primary objective of this study was to perform a retrospective analysis of the overall prevalence of SN among orthodontic patients compared with non-orthodontic controls in both pediatric and adult populations using an existing salivary biorepository.

## 2. Materials and Methods

### 2.1. Protocol Review

This retrospective study was conducted in accordance with the Declaration of Helsinki, using an approved protocol, which was reviewed by the Office for the Protection of Research Subjects (OPRS) and the Institutional Review Board (IRB) for the University of Nevada Las Vegas (UNLV). This retrospective analysis was deemed “Research Exempt”, as defined by the United States Department of Health and Human Services (US-DHHS) 45 Code of Federal Regulations (CFR) 46 that declares that research involving existing biorepository samples that are not prospectively collected and have no patient-specific or patient-identifiable information is exempt from collecting Informed Consent. The protocol 1717625-1 titled “Retrospective analysis of microbial prevalence from DNA isolated from saliva samples originally obtained from the University of Nevada, Las Vegas (UNLV) School of Dental Medicine (SDM) pediatric and clinical population” was approved on 3 March 2021.

### 2.2. Original Sampling Protocol

The collection of clinical saliva samples was originally reviewed and approved by the UNLV IRB and OPRS under the protocol titled “The Prevalence of Oral Microbes in Saliva from the University of Nevada, Las Vegas School of Dental Medicine (UNLV-SDM) Pediatric Adult Clinical Population” 1305-4466M. Inclusion criteria: The samples were collected only from voluntary participants that were patients of record at UNLV-SDM and were willing to provide Informed Consent if aged 18 years or older and Pediatric Assent if aged under 18 with the consent of the parent or guardian (inclusion criteria). Exclusion criteria: Any patient that was invited to participate could decline to participate or could be excluded if they refused to provide Informed Consent or Pediatric Assent. Any person who was not a patient of record at UNLV-SDM was also excluded.

Briefly, unstimulated saliva samples were previously collected on randomly selected days, using sterile 50 mL collection tubes labeled with a randomly generated, non-duplicated number to prevent any patient identifying information from being collected. Basic demographic (non-identifying) information was simultaneously collected, which included the patient’s age in years, sex, orthodontic status, and any racial or ethnic information they chose to divulge. Samples were transferred to a secured biomedical laboratory for processing and immediate storage at −80 °C.

### 2.3. DNA Isolation

The isolation of DNA from saliva samples was previously performed using the TRIzol phenol–chloroform extraction reagent obtained from Invitrogen (Waltham, MA, USA). Samples were thawed and mixed prior to placing 500 μL of sample with 500 μL of TRIzol and 200 μL of chloroform in a sterile microcentrifuge tube. Following a brief incubation on ice, each sample was subsequently centrifuged at 10,000× *g* relative centrifugal force (RCF) using an Eppendorf 5425 R Microcentrifuge (Hamburg, Germany) for 15 min. The upper phase was removed and mixed with isopropanol in a fresh microcentrifuge tube to precipitate the DNA prior to centrifugation at the same speed for ten minutes. The isopropanol was removed, and the pellet was then washed with ethanol prior to a final centrifugation at the same speed for five minutes. The ethanol was aspirated, and the DNA pellet was resuspended using nuclease-free water. Quantification and determination of purity was performed using a NanoDrop 2000 spectrophotometer from Fisher Scientific (Fair Lawn, NJ, USA) and the protocol recommended by the manufacturer [34,35].

### 2.4. Screening Protocol

DNA previously isolated from patient samples was screened for the presence of SN using the QuantStudio real-time polymerase chain reaction (PCR) system from Applied Biosystems (Waltham, MA, USA). In brief, samples were prepared for analysis using the Power Track SYBR Green Master Mix also from Applied Biosystems (Waltham, MA, USA), as previously described [24,25]. Each reaction was performed in duplicate using 2.0 μL of sample DNA diluted to a concentration of 100 ng/mL, 1.5 μL each of forward and reverse primer, 7.5 μL of nuclease-free water, and 12.5 μL of 2X Power Track SYBR Green Master Mix for a total volume of 25 μL. Reactions were performed using the recommended protocol, which involved one cycle of 95 °C for 20 s of enzyme activation and 40 cycles of 60 °C for 30 s for primer annealing and PCR extension interspersed with five seconds at 95 °C. Data were then exported into Microsoft Excel (Redmond, WA, USA) for analysis. All samples were screened for the presence of human DNA and bacterial DNA (positive controls) and SN (experimental) using the following validated primers [33,34,35]:
Human DNA-Positive controlBeta actin forward primer,5′-GTGGGGTCCTGTGGTGTG-3′Beta actin reverse primer,5′-GAAGGGGACAGGCAGTGA-3′Bacterial DNA-Positive control16S rRNA forward primer,5′-ACGCGTCGACAGAGTTTGATCCTGGCT-3′16S rRNA reverse primer,5′-GGGACTACCAGGGTATCTAAT-3′SN primersSN forward primer,5′-TCTGGGCTACACACGTACTACAATG-3′SN reverse primer,5′-GCCTGCAATCCGAACTGAGA-3′

### 2.5. Data Analysis

Demographic variables were organized and summarized using descriptive statistics and reported as total numbers and percentages of the total study sample. Statistical analysis comparing the study sample to the clinic population was completed using Chi-square analysis, which allows for comparisons of non-parametric (categorical) data. Screening results (SN-positive and SN-negative) were also analyzed with Chi-square analysis, using the GraphPad Prism (Version 9) online software package (San Diego, CA, USA).

## 3. Results

The overall study sample consisted of *n* = 208 samples, which were equally divided between males (50%) and females (50%) and similar to the overall clinic patient population, *p* = 0.8414 (Table 1). A majority of patients were minority or non-White (66.8%), which was also similar to the overall patient population (70.4%), *p* = 0.5127, with most of those minority patients self-identified as Hispanic or Latino. Finally, the average age of the study sample was 23.9 years (range from 0 to 69 years), which was similar to the overall clinical population of 25.7 years (range 0 to 89 years), *p* = 0.0441.

A more detailed analysis revealed that the pediatric study sample consisted of a total of *n* = 89 samples, which were nearly equally divided between males (49.4% or *n* = 44/89) and females (50.6% or *n* = 45/89) that closely matched the overall percentages of males and females from the overall clinic patient population, *p* = 0.6886 (Table 2). The analysis of race and ethnicity data demonstrated that samples from White or Caucasian and non-White or minority patients accounted for 34.8% and 65.2% of the study, respectively, which was different from the overall clinic patient population, with 24.7% White and 75.3% minority patients, respectively (*p* = 0.0209). Most of the minority patients in the study sample (31.5%) and the clinic population (52.4%) were Hispanic or Latino, with smaller percentages identifying as Black or African American (16.9% and 12.2%, respectively) or Asian/Pacific Islander (11.2% and 3.8%, respectively). Study-sample ages ranged from 7 to 18 years, with an average age of 13.5 years, which was higher than the overall clinic patient population of 9.04 years, with an age range that extended from 0 to 18 years, *p* = 0.0221.

The adult study sample consisted of *n* = 119 samples, which consisted of approximately equal proportions of males (50.4% or *n* = 60/119) and females (49.6% or *n* = 59/119) and closely matched the overall adult clinic patient population demographics (49.1% and 50.9%, respectively), *p* = 0.8414 (Table 3). Our assessment of the self-reported race and ethnicity information revealed that the proportion of samples from White or Caucasian patients (31.9% or *n* = 38/119) was similar to the overall adult clinic population (34.6%), *p* = 0.5294. Most of the samples were derived from minority patients (68.1% or *n* = 81/119), which was also similar to the overall clinic (65.4%), with the overwhelming majority of those identified as Hispanic or Latino (44.5% or *n* = 53/119 and 49.4%, respectively) and smaller percentages of Black or African American (16.8% or *n* = 20/119 and 12.2%, respectively) or Asian or Pacific Islander (3.4% or *n* = 4/119 and 3.8%, respectively). The age of adult patients in the study sample averaged 34.3 years, with a range of 17 to 69 years, and this average was significantly lower than the overall adult clinic population average of 42.3 years, which ranged between 18 and 89 years, *p* = 0.0229.

**Figure 1:**
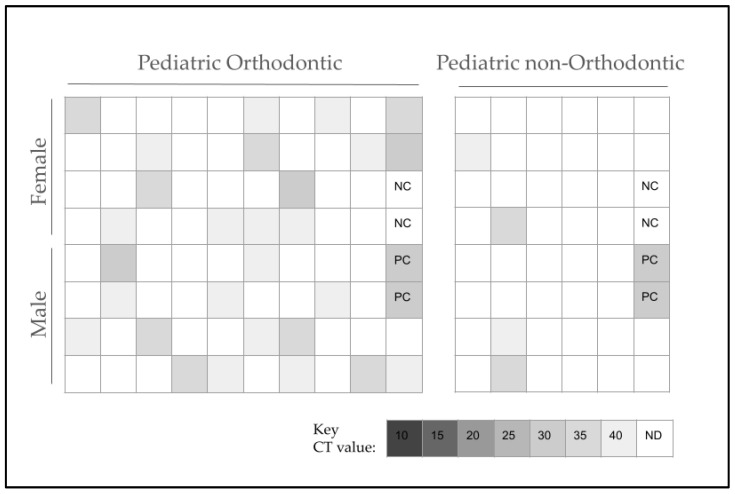
Pediatric sample screening for SN. More than one-third, or 35.9% (*n* = 32/89), of pediatric samples harbored SN, while 64.1%, or *n* = 57/89, were SN-negative, and the samples were equally divided among males (53.1% or *n* = 17/32) and females (46.9% or *n* = 15/32). Most SN-positive samples were minority patients (62.5% or *n* = 20/32) compared with White or Caucasian patients (37.5% or *n* = 12/32), an outcome that was similar to the overall study sample (65.2% or *n* = 58/89 and 34.8% or *n* = 31/89, respectively). SN-positive samples were more prevalent among pediatric orthodontic patients (87.5% or *n* = 28/32) versus non-orthodontic patients (12.5% or *n* = 4/32), an observation that was different than the overall proportion of orthodontic versus non-orthodontic pediatric study samples (53.9% or *n* = 48/89 and 46.1% or *n* = 41/89, respectively), *p* = 0.0001. NC = negative control, PC = positive control, (qPCR) CT = cycle threshold value, ND = not detected.

Pediatric samples (*n* = 89) were screened using validated primers for SN (Figure 1). This screening revealed that 35.9% or *n* = 32/89 of pediatric samples harbored SN, while 64.1% or *n* = 57/89 were SN-negative. Among the SN-positive samples, these were nearly equally divided between males (53.1% or *n* = 17/32) and females (46.9% or *n* = 15/32), making them similar to the overall distribution of males and females in the study sample (49.4% or *n* = 44/89 and 50.6% or *n* = 45/89, respectively). In addition, most of the SN-positive samples were from minority patients (62.5% or *n* = 20/32) compared with White or Caucasian patients (37.5% or *n* = 12/32), an observation similar to the overall distribution of minority and White patients in the overall study sample (65.2% or *n* = 58/89 and 34.8% or *n* = 31/89, respectively). However, a greater proportion of SN-positive samples were found among pediatric orthodontic patients (87.5% or *n* = 28/32) versus non-orthodontic patients (12.5% or *n* = 4/32), so the proportion was different than the proportion among orthodontic versus non-orthodontic pediatric samples (53.9% or *n* = 48/89 and 46.1% or *n* = 41/89, respectively), *p* = 0.0001.

To more accurately assess any potential differences between SN-positive and SN-negative samples, each of the available demographic characteristics was evaluated (Table 4). This analysis demonstrated that although the proportion of males and females among the SN-positive samples (53.1% or *n* = 17/32 and 46.9% or *n* = 15/32) was similar to the overall pediatric clinic population (49.4% or *n* = 44/89 and 50.6% or *n* = 45/89, respectively), it was also similar among the SN-negative samples (47.4% or *n* = 27/57 and 52.6% or *n* = 30/57), a result that was not statistically significant, *p* = 0.2293. Our evaluation of race and ethnicity confirmed that a majority of SN-positive (62.5% or *n* = 20/32) and SN-negative samples (66.7% or *n* = 38/57) were from minority patients, making the distribution similar to the overall distribution of minority patients in the overall study sample (65.2% or *n* = 58/89), *p* = 0.2876. Although a majority of pediatric study sample patients were undergoing orthodontic therapy at the time of sampling, a greater proportion of SN-positive samples were found among the orthodontic patients (87.5% or *n* = 28/32) than non-orthodontic patients (12.5% or *n* = 4/32), so the proportion was significantly greater than that observed with SN-negative orthodontic (78.9% or *n* = 45/57) and non-orthodontic (21.1% or *n* = 12/57) patients, *p* = 0.0271. 

**Figure 2:**
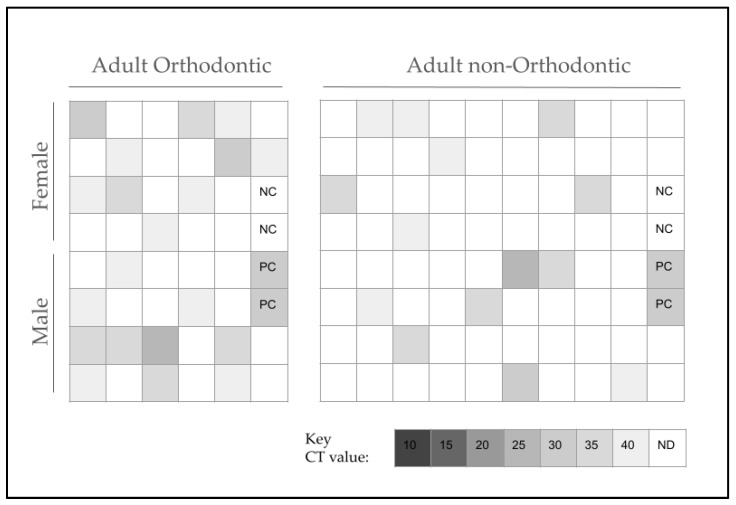
Adult sample screening for SN. Approximately one-third, or 28.6% (*n* = 34/119), of adult samples harbored SN, while 71.4%, or *n* = 85/119, were SN-negative, which was divided nearly equally among males (52.9% or *n* = 18/34) and females (47.1% or *n* = 16/34). Most SN-positive adult samples were also from non-White or minority patients (73.5% or *n* = 25/34), which was similar to the overall adult study sample (68.1% or *n* = 81/119), *p* = 0.1984. SN-positive adult samples were more prevalent among orthodontic patients (58.8% or *n* = 20/34) than the overall proportion of adult orthodontic study samples (36.9% or *n* = 44/119), *p* = 0.0001. NC = negative control, PC = positive control, (qPCR) CT = cycle threshold value, ND = not detected.

Adult samples (*n* = 119) were also screened for SN using the same protocol (Figure 2). This analysis demonstrated that 28.6% or *n* = 34/119 harbored SN, with 71.4% or *n* = 85/119 testing negative. A further evaluation of the SN-positive samples revealed no significant differences between the percentage of SN-positive males (52.9% or *n* = 18/34) and females (47.1% or *n* = 16/34) and the overall adult study sample (50.4% and 49.6%, respectively), *p* = 0.4236. Similar to the pediatric patients, most of the SN-positive samples within the adult study sample were also derived from minority patients (73.5% or *n* = 25/34), and this percentage was similar to the percentage of minorities in the overall adult study sample (68.1% or *n* = 81/119), *p* = 0.1984. However, a significantly higher proportion of SN-positive samples was found among adult orthodontic patients (58.8% or *n* = 20/34) versus non-orthodontic patients (41.2% or *n* = 14/34), and this proportion was greater than the overall proportion of adult orthodontic (36.9% or *n* = 44/119) and non-orthodontic (63.1% or *n* = 75/119) samples, *p* = 0.0001.

To evaluate any potential differences between SN-positive and SN-negative adult samples, all demographic variables were analyzed in detail (Table 5). These results demonstrated that the proportion of adult males and females among the SN-positive samples (52.9% or *n* = 18/34 and 47.1% or *n* = 16/34) was similar to that of the SN-negative samples (49.4% or *n* = 42/85 and 50.6% or *n* = 43/85), and the proportion of adult males and females was not significantly different from that of the overall adult study sample (50.4% and 49.6%, respectively), *p* = 0.4236. In addition, most adult SN-positive (73.5% or *n* = 25/34) and SN-negative samples (65.9% or *n* = 56/85) were from minority patients, and this distribution was similar to the overall distribution of minority patients in the overall adult study sample (68.1% or *n* = 81/119), *p* = 0.0913. Finally, although few adult study-sample patients were undergoing orthodontic therapy at the time of sampling, a greater proportion of SN-positive samples was found among the orthodontic patients (58.8% or *n* = 20/34) than non-orthodontic patients (41.2% or *n* = 14/34), and this proportion was significantly greater than that observed with SN-negative orthodontic (28.2% or *n* = 24/85) and non-orthodontic (71.8% or *n* = 61/85) patients, *p* = 0.0001.

## 4. Discussion

The overall purpose of this study was to evaluate the oral prevalence of SN to determine if any differences could be found between orthodontic and non-orthodontic patient samples from an existing biorepository. The key findings of this study revealed that approximately one-third of all samples (including both pediatric and adult) harbored DNA specific for SN, which was also found to be significantly higher among samples from orthodontic patients compared with non-orthodontic patients regardless of age. These data confirm other results from recent prevalence studies that suggest SN may be present in both pediatric and adult patient populations in roughly similar proportions [34,35,40]. Moreover, these studies (in conjunction with these current results) represent the majority of oral prevalence data regarding SN that does not come exclusively from older adult or elderly patient populations [41,42,43].

In addition, these data confirm previous observations that associations were found with patient sex and the oral presence of SN, although many other periodontal pathogens appear to be highly influenced by sex and the associated influence of hormones on periodontal disease development and progression [34,35,44,45]. Many studies have demonstrated that sex and associated sex-specific hormones may have significant impacts on biofilm formation on mucosal surfaces within the oral cavity and gastrointestinal tract [46,47,48]. However, these findings provide additional data that SN may be one of the few periodontal pathogens that does not exhibit this type of sex-specific prevalence, thus providing further evidence of the significance of this prevalence study regarding this microbe.

The most significant association observed from these results may be that clinical samples from orthodontic patients exhibited a higher prevalence of SN than for non-orthodontic patients, thus adding to the extremely limited number of studies that have evaluated this phenomenon [20,21,39]. Moreover, these findings also evaluated both pediatric and adult patients with and without orthodontic appliances, which demonstrated that these differences were observed among both adolescent patients and younger adults with brackets, suggesting that this variable may be much more significant towards understanding the oral prevalence of this microorganism than was previously understood [49,50,51]. However, these results also confirmed that the largest differences in oral SN prevalence were observed between adult orthodontic and adult non-orthodontic patients, suggesting that at least some of the variance observed may have an age-related component that will need further analysis for a more accurate understanding of these observations [34,35].

These findings also confirm the important link between the placement of orthodontic brackets and the development of other conditions, such as periodontal disease, that may have lasting or long-term impacts on patient health and disease development [52,53]. For example, although much is known about the development of gingivitis and periodontitis among patients with orthodontic brackets, these conditions are almost always reversible with the removal of brackets at the end of treatment [54,55,56]. The new understanding that SN may lead to long-term health consequences, such as increased weight and body mass index with the associated complications of metabolic syndrome and other systemic diseases, may alter how oral-health researchers and clinical scientists view the importance of preventing and eliminating gingivitis and periodontal disease development among adolescent and younger patients undergoing orthodontic treatment [32,34,36,37,38].

Despite the significance and importance of these findings, there are some limitations associated with this type of research that should also be considered. For instance, this study involved a retrospective analysis of existing clinical samples and may not reflect the most current prevalence and epidemiology of SN [21,34,35]. Prospective studies that derive samples from patients following the SARS-CoV-2 (COVID-19) pandemic may reveal differences in oral microbial prevalence due to delayed treatment or reduced clinical capacities that persist among many patient populations [40,57]. In addition, the increasing availability and popularity of alternative forms of orthodontic treatment, such as clear aligners and other non-bracket alternatives, may also impact the development of periodontal disease or the prevalence of periodontal pathogens; however, no samples from patients with clear aligners were available within the biorepository for the current study [58,59,60]. Moreover, although this information was not available in this retrospective analysis, additional factors, such as the length of time the brackets or appliances were in place prior to sampling, may also be an important factor that should be more closely evaluated in future studies to determine any temporal associations with SN prevalence during orthodontic treatment [54,55,56]. Finally, due to the retrospective nature of this study, only limited demographic data were available to the research team that do not allow for the evaluation of additional variables, such as brushing frequency, flossing, or electric toothbrush use in this patient population, but may be considerations for other researchers that would like to assess methods to reduce the prevalence of SN in future studies involving this microbe [61,62,63,64].

## 5. Conclusions

The data from this study confirm that no sex-specific differences were observed in the prevalence of SN, but that significant differences were found between orthodontic and non-orthodontic patients that also varied according to the age of the patient. These data suggest that both pediatric orthodontic patients exhibited a higher prevalence of SN compared with their age-matched non-orthodontic controls, but these differences were much more significant among young adults versus adolescents. Due to the association of this microorganism not only with periodontal disease but with other long-term health issues, such as obesity, further research will be needed to understand the many complicating factors that influence and modulate the prevalence of this microbe among patient populations and to design protocols and interventions that reduce the oral burden of SN among affected patients.

## Figures and Tables

**Table 1:** Overall study-sample demographics.

Demographic	Overall Study Sample (*n* = 208)	Overall Clinic Patient Population	Statistical Analysis
**Sex**			
Male	50.0% (*n* = 104/208)	48.1%	X^2^ = 0.040, d.f. = 1 *p* = 0.8414
Female	50.0% (*n* = 104/208)	51.9%	
**Race/Ethnicity**			
White/Caucasian	33.2% (*n* = 69/208)	29.6%	X^2^ = 0.429, d.f. = 1 *p* = 0.5127
Non-White/minority	66.8% (*n* = 139/208)	70.4%	
Hispanic or Latino	38.9% (*n* = 81/208)	50.9%	
Black or African American	16.8% (*n* = 35/208)	12.2%	
Asian or Pacific Islander	6.7% (*n* = 14/208)	7.5%	
**Age**			
Average	23.9 years	25.7 years	Two-tailed *t*-test *p* = 0.0441
Range	0 to 69 years	0 to 89 years	

**Table 2:** Demographic characteristics of pediatric study sample.

Demographic	Pediatric Study Sample (*n* = 89)	Pediatric Clinic Patient Population	Statistical Analysis
**Sex**			
Male	49.4% (*n* = 44/89)	47.2%	X^2^ = 0.161, d.f. = 1 *p* = 0.6886
Female	50.6% (*n* = 45/89)	52.8%	
**Race/Ethnicity**			
White/Caucasian	34.8% (*n* = 31/89)	24.7%	X^2^ = 5.333, d.f. = 1 *p* = 0.0209
Non-White/minority	65.2% (*n* = 58/89)	75.3%	
Hispanic or Latino	31.5% (*n* = 28/89)	52.4%	
Black or African American	16.9% (*n* = 15/89)	12.2%	
Asian or Pacific Islander	11.2% (*n* = 10/89)	3.8%	
**Age**			
Average	13.5 years	9.04 years	Two-tailed *t*-test *p* = 0.0221
Range	7 to 18 years	0 to 17 years	

**Table 3:** Demographic characteristics of adult study sample.

Demographic	Adult Study Sample (*n* = 119)	Adult Clinic Patient Population	Statistical Analysis
**Sex**			
Male	50.4% (*n* = 60/119)	49.1%	X^2^ = 0.040, d.f. = 1 *p* = 0.8414
Female	49.6% (*n* = 59/119)	50.9%	
**Race/Ethnicity**			
White or Caucasian	31.9% (*n* = 38/119)	34.6%	X^2^ = 0.396, d.f. = 1 *p* = 0.5294
Non-White/minority	68.1% (*n* = 81/119)	65.4%	
Hispanic	44.5% (*n* = 53/119)	49.4%	
Black or African American	16.8% (*n* = 20/119)	12.2%	
Asian or Pacific Islander	3.4% (*n* = 4/119)	3.8%	
**Age**			
Average	34.3 years	42.3 years	Two-tailed *t*-test *p* = 0.0229
Range	17 to 69 years	18 to 89 years	

**Table 4:** Analysis of SN qPCR screening results—pediatric.

Demographic	SN-Positive (*n* = 32)	SN-Negative (*n* = 57)	Statistical Analysis
**Sex**			
Males	53.1% (*n* = 17/32)	47.4% (*n* = 27/57)	X^2^ = 1.445, d.f. = 1 *p* = 0.2293
Females	46.9% (*n* = 15/32)	52.6% (*n* = 30/57)	
**Race/Ethnicity**			
White/Caucasian	37.5% (*n* = 12/32)	33.3% (*n* = 19/57)	X^2^ = 1.131, d.f. = 1 *p* = 0.2876
Non-White/minority	62.5% (*n* = 20/32)	66.7% (*n* = 38/57)	
**Orthodontic status**			
Orthodontic brackets	87.5% (*n* = 28/32)	78.9% (*n* = 45/57)	X^2^ = 4.882, d.f. = 1 *p* = 0.0271
No orthodontic brackets	12.5% (*n* = 4/32)	21.1% (*n* = 12/57)	

**Table 5:** Analysis of SN qPCR screening results for adults.

Demographic	SN-Positive (*n* = 34)	SN-Negative (*n* = 85)	Statistical Analysis
**Sex**			
Males	52.9% (*n* = 18/34)	49.4% (*n* = 42/85)	X^2^ = 0.640, d.f. = 1 *p* = 0.4236
Females	47.1% (*n* = 16/34)	50.6% (*n* = 43/85)	
**Race/Ethnicity**			
White/Caucasian	26.5% (*n* = 9/34)	34.1% (*n* = 29/85)	X^2^ = 2.852, d.f. = 1 *p* = 0.0913
Non-White/minority	73.5% (*n* = 25/34)	65.9% (*n* = 56/85)	
**Orthodontic status**			
Orthodontic brackets	58.8% (*n* = 20/34)	28.2% (*n* = 24/85)	X^2^ = 47.669, d.f. = 1 *p* = 0.0001
No orthodontic brackets	41.2% (*n* = 14/34)	71.8% (*n* = 61/85)	

## Data Availability

The primary data may be available upon request from the corresponding author. These data are not publicly available according to the protection parameters for the study protocol, which were required by the IRB and the Office for the Protection of Research Subjects (OPRS) for the study approval.
